# Thermo-Responsive Graphene Oxide/Poly(Ethyl Ethylene Phosphate) Nanocomposite via Ring Opening Polymerization

**DOI:** 10.3390/nano9020207

**Published:** 2019-02-05

**Authors:** Xue Jiang, Guolin Lu, Xiaoyu Huang, Yu Li, Fangqi Cao, Hong Chen, Wenbin Liu

**Affiliations:** 1 Shanghai Key Laboratory of Crime Scene Evidence, Shanghai Research Institute of Criminal Science and Technology, Zhongshan North No 1 Road, Shanghai 200083, China; 13795305289@163.com (X.J.); 13675113399@163.com (Y.L.); frankie-cao@163.com (F.C.); chenhong2898@163.com (H.C.); 2 Key Laboratory of Synthetic and Self-Assembly Chemistry for Organic Functional Molecules, Shanghai Institute of Organic Chemistry, Chinese Academy of Sciences, 345 Lingling Road, Shanghai 200032, China; luguolin@mail.sioc.ac.cn

**Keywords:** graphene oxide, PEEP, ROP, grafting-from

## Abstract

An efficient strategy for growing thermo-sensitive polymers from the surface of exfoliated graphene oxide (GO) is reported in this article. GO sheets with hydroxyls and epoxy groups on the surface were first prepared by modified Hummer’s method. Epoxy groups on GO sheets can be easily modified through ring-opening reactions, involving nucleophilic attack by tris(hydroxymethyl) aminomethane (TRIS). The resulting GO-TRIS sheets became a more versatile precursor for next ring opening polymerization (ROP) of ethyl ethylene phosphate (EEP), leading to GO-TRIS/poly(ethyl ethylene phosphate) (GO-TRIS-PEEP) nanocomposite. The nanocomposite was characterized by ^1^H NMR, Fourier transform infrared spectroscopy (FT-IR), X-ray photoelectron spectroscopy (XPS), thermogravimetric analysis (TGA), differential thermal gravity (DTG), transmission electron microscopy (TEM) and atomic force microscopy (AFM). Since hydrophilic PEEP chains make the composite separate into single layers through hydrogen bonding interaction, the dispersity of the functionalized GO sheets in water is significantly improved. Meanwhile, the aqueous dispersion of GO-TRIS-PEEP nanocomposite shows reversible temperature switching self-assembly and disassembly behavior. Such a smart graphene oxide-based hybrid material is promising for applications in the biomedical field.

## 1. Introduction

Graphene oxide (GO), a single layer of carbon atoms in a closely packed honeycomb two-dimensional lattice with carboxylic acid, epoxide, and hydroxyl groups, has attracted considerable attention in recent years [1,2,3,4,5,6,7,8,9]. GO, which is highly hydrophilic, can be prepared using cheap graphite as raw material by cost-effective chemical methods with a high yield. The biocompatibility of GO renders it a good candidate for application in the biomedical field [10,11,12,13]. The presence of abundant functional groups at the surface of GO may be very interesting since that they provide enough reactive sites for the subsequent chemical modification using known carbon surface chemistry [14]. Recently, a considerable number of works have been performed on enhancing the properties of GO by adding additional functionality to the groups already present on the surface of GO [15,16,17,18,19,20,21,22,23,24,25,26,27,28]. In particular, “intelligentizing” graphene can been obtained by attaching stimuli-responsive polymers to the backbone of GO [21,22,23,24,25,26,27,28]. These attachments are typically made by either grafting-from or -onto approaches. The vast majority of the triggers reported so far are confined to temperature [21,22,23,24], pH [24,25], light [26,27] and redox [28].

Polyphosphoesters (PPEs) are known to be a class of biocompatible polymers with repeated phosphoester attachments in the backbone [29,30,31,32,33]. As the phosphorus atom has multivalent property, the physicochemical properties can be easily modified by means of introduction into such polymers with different functional groups. According to the type of side groups connected to the phosphorous atom, PPEs are classified as polyphosphate, polyphosphonate, polyphosphite or polyphosphoramidate [33]. Among them, polyphosphates are an important family of PPEs, which have attracted great attention for biological and phamaceutical applications such as drug release, gene transfer, and tissue engineering [34,35,36,37]. Under usual physiological conditions, polyphosphates can readily degrade through hydrolysis or enzymatic cleavage of the phosphoester bonds. In addition to the excellent biocompatibility and biodegradability, it has been demonstrated that polyphosphates also exhibit thermo-responsibility in aqueous solution so as to make them possess great potential as novel smart biomaterials [32,38]. However, no one has reported on a PPE-based GO/polymer nanocomposite until now.

**Scheme 1:**
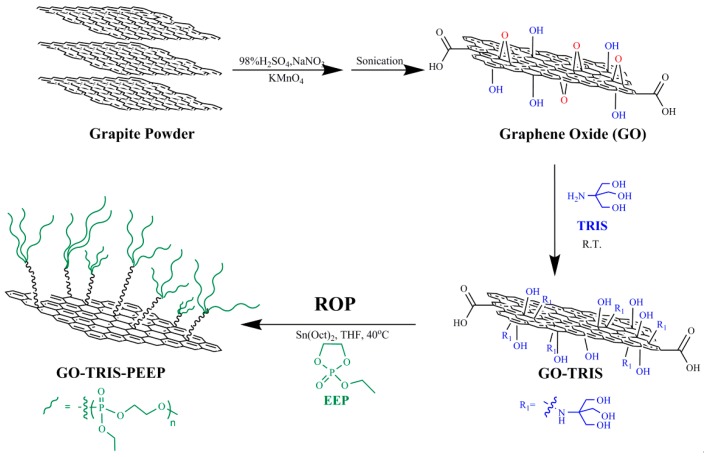
Preparation of graphene oxide-tris(hydroxymethyl) aminomethane-poly(ethyl ethylene phosphate) (GO-TRIS-PEEP) nanocomposite.

Herein, we have successfully designed and prepared a kind of PPE-based temperature- responsive GO/polymer nanocomposite (Scheme 1), in which a typical hydrophilic polyphosphoester, i.e., poly(ethyl ethylene phosphate) (PEEP), was covalently connected on the surface of GO by ring opening polymerization (ROP) of ethyl ethylene phosphate (EEP) initiated by the hydroxyls on GO surface introduced by tris(hydroxymethyl) aminomethane (TRIS). The obtained GO-TRIS-PEEP nanocomposite has robust temperature-responsive properties resulting from a change in the PEEP conformation on GO surface. When raising the temperature, PEEP chains may become hydrophobic and collapse so that the nanocomposite precipitated from the solution. Owing to the excellent biocompatibility, biodegradability and thermo-responsibility of PEEP chains, the prepared nanocomposite shows a bright prospect in the field of smart nanocarrier for controlled release under temperature stimuli.

## 2. Experimental

### 2.1.Characterization

^1^H NMR measurements were performed on a JEOL resonance ECZ 400S (400 MHz) spectrophotometer (Tokyo, Japan) in CDCl_3_ or D_2_O, TMS was used as internal standard. Fourier transform infrared (FT-IR) spectra were recorded on a Nicolet AVATAR-360 FT-IR spectrophotometer (Waltham, MA, USA) with a resolution of 4 cm^−1^. Elemental analysis was carried out on a Carlo-Erba 1108 system (Milan, Italy). X-ray photoelectron spectroscopy (XPS) was recorded on a Perkin Elmer PHI 5000c ESCA photoelectron spectrometer (Waltham, IN, USA). Thermogravimetric analysis (TGA) was carried out with a TA Q500 thermal analyzer (New Castle, IN, USA) from 50 °C to 600 °C at a heating rate of 10 °C/min in N_2_. Transmission electron microscopy (TEM) images were obtained by a JEOL JEM 1230 instrument (Tokyo, Japan) operated at 80 kV. Atomic force microscopy (AFM) images were taken by a Veeco DI MultiMode SPM (Plainview, TX, USA) in the tapping mode of dropping the sample solution onto the freshly exfoliated mica substrate. Differential scanning calorimetry (DSC) measurement was run on a TA Q200 (New Castle, IN, USA) system under N_2_ purge with a heating rate of 10 °C min^−1^.

### 2.2. Preparation of GO Sheets

Exfoliated GO sheets were prepared by a modified Hummer’s method using graphite powder as starting material. Graphite powder (99.99+%, Aldrich) was firstly oxidized by sulfuric acid (H_2_SO_4_, Aldrich, 95–98%) and potassium permanganate (KMnO_4_, Aldrich, 99%) followed by filtration and subsequent dialysis or by several runs of centrifugation/washing to completely remove residual salts and acids. Finally, graphite oxide dispersion (0.1 mg mL^−1^) was exfoliated by waterbath ultrasonication for 3 h. GO sheets were recovered by filtration and vacuum drying.

### 2.3. Preparation of TRIS-Bonded Graphene Sheets (GO-TRIS)

GO (2.3 g) was treated with 12.2 g of 1,1,1-tris(hydroxymethyl) methanamine (TRIS, Aldrich, 99%) in 500 mL of anhydrous *N*,*N*-dimethylformamide (DMF, Aldrich, 99.8%) at room temperature for 36 h to transform the epoxy groups on the basal plane of GO into hydroxyls. The crude product was filtered through a 0.22 μm filter and washed exhaustively with deionized water. The obtained GO-TRIS (2.4 g) was dried overnight at 40 °C in vacuo.

### 2.4. Preparation of Ethyl Ethylene Phosphate (EEP)

Tetrahydrofuran (THF, Aldrich, 99%) was dried over CaH_2_ and distilled from sodium and benzophenone under N_2_ prior to use. Triethylamine (Et_3_N, Aldrich, 99.5%) was dried over KOH and distilled over CaH_2_ under N_2_ prior to use. A mixture of ethanol (EtOH, Aldrich, 99.8%, 8.8 mL, 0.15 mol) and Et_3_N(20.9 mL, 0.15 mol) was added dropwise to a solution of 2-chloro-1,3,2- dioxaphospholane-2-oxide (COP, Aldrich, 90%–94%, 21.4 g, 0.15 mol) in anhydrous THF (250 mL) at −5 °C. The reaction mixture was stirred at −5 °C for 30 min and then at room temperature for additional 12 h. The resulting mixture was firstly filtered to remove the insoluble salt. The filtrate was concentrated and distilled under reduced pressure (107 Pa/95~97 °C) to give 15.5 g of yellowish liquid, ethyl ethylene phosphate (EEP), with a yield of 68%. ^1^H NMR (400 MHz, CDCl_3_): *δ* (ppm): 1.38 (3H, POCH_2_C*H*_3_), 4.22 (2H, POC*H*_2_CH_3_), 4.42 (4H, OC*H*_2_C*H*_2_).

### 2.5. Preparation of PEEP-Grafted Graphene Sheets (GO-TRIS-PEEP)

Tin-2-ethylhexanoate (Sn(Oct)_2_, Aldrich, 98%) was purified by distilling under reduced pressure (20~40 Pa/152 °C) after co-boiling with *p*-xylene twice prior to use [39]. A 25 mL Schlenk flask was first dried by sonication in acetone. It was then immersed in the diethyl ether solution of trimethylchlorosilane (5% TMSCl) overnight to remove the hydroxyls on the surface of glass. GO-TRIS (235 mg) was firstly added to the flask (flame-dried under vacuum prior to use) sealed with a rubber septum for degassing and kept under Ar. After three cycles of evacuation purging with purified Ar, anhydrous THF (15 mL) and DMF (2 mL) were added via a gastight syringe followed by water-bath ultra-sonication for 30 minutes. The flask was then degassed by three cycles of freezing-pumping-thawing after EEP (1.98 g, 13 mmol) was added. THF solution (0.9 mL) of Sn(Oct)_2_ (0.18 mmol) was added via a gastight syringe. The flask was again degassed by three cycles of freezing-pumping-thawing, followed by immersing the flask into an oil bath set at 40 °C. The polymerization lasted 24 h and it was terminated by adding excess acetic acid. The crude product was filtered through a 0.22 μm filter followed by washing with THF and deionized water. The precipitate was dried *in* *vacuo* at 45 °C for 48 h to give the desired product of GO-TRIS-PEEP (156 mg). XPS (molecular molar ratio): C, 65.66%; N, 0.98%; O, 30.44%, P, 1.89%. ^1^H NMR (400 MHz, D_2_O): *δ* (ppm): 1.12 (3H, POCH_2_C*H*_3_), 3.81 (2H, POC*H*_2_CH_3_), 4.16 (4H, OC*H*_2_C*H*_2_). FT-IR: *ν* (cm^–1^): 1727, 1590, 1226, 1030, 977, 800, 640, 518.

## 3. Results and discussion

### 3.1. Preparation of GO-TRIS-PEEP

**Figure 1:**
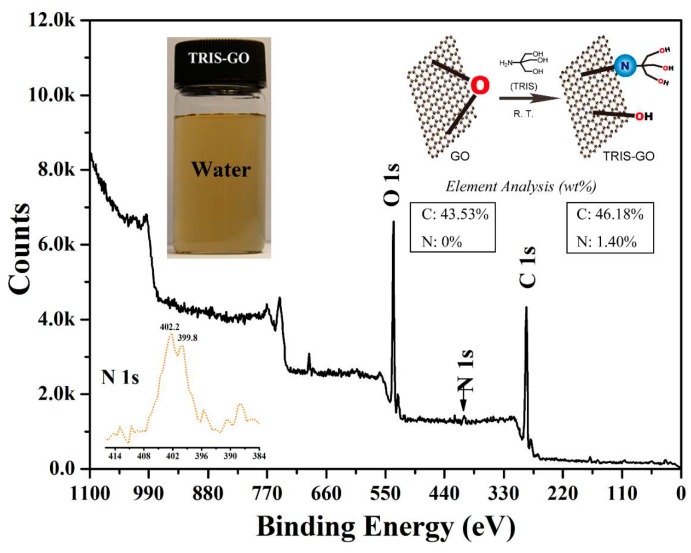
Survey X-ray photoelectron spectroscopy (XPS) data for GO-TRIS and photograph of well-dispersed GO-TRIS solution in water (inset).

The fabrication process of GO-TRIS-PEEP polymer-modified nanocomposite via ring opening polymerization of EEP monomer is outlined in Scheme 1**.** Commercial graphite powder was first oxidized to GO with the help of KMnO_4_ under strong acidic environment using the modified Hummer’s method [40], as a result, new epoxy and hydroxyl functionalities were introduced to the surface of a graphene oxide sheet. The structure of the obtained GO was confirmed by FT-IR (Figure S1), XRD (Figure S2) and elementary analysis (Table S1). The obtained epoxy groups can be easily modified through ring-opening reactions, on the basis of the mechanism of nucleophilic attack by amine groups [17]. 1,1,1-Tris(hydroxymethyl) methanamine (TRIS) is a kind of primary amine with three hydroxyls, which has been extensively used in chemical modification of macroporous materials [41,42]. In this work, TRIS is required to improve the opportunity of grafting from the plane of GO sheet, rendering GO a more versatile precursor for a wide range of applications. XPS measurement was used to confirm the introduction of TRIS moiety. XPS spectrum of GO-TRIS shown in Figure 1 exhibits a significant nitrogen peak around the binding energy of 400 eV, which is absent in that of GO (Figure S3). Zooming in further revealed that the N1s band appeared at 402.2 eV accompanied with a lower binding energy shoulder located at 399.8 eV, which is consistent with a previous report [43]. The appearance of N1s band suggested the successful covalent functionalization of TRIS units onto the surface of modified GO sheets; i.e., the formation of TRIS-modified GO (GO-TRIS). Moreover, Figure 1 also displays the elemental analysis result of GO-TRIS, which indicated that the nitrogen content (N%) increased from 0 to 1.40% after the functionalization with TRIS. The C/N ratio was estimated to be 38 = (46.18wt %/12)/(1.40 wt %/14) (1 TRIS group per 6.3 aromatic rings), which agreed with the value (45) derived from XPS measurement. Additionally, the dispersity of GO-TRIS kept as good as GO in water or polar organic solvents as shown in the inset of Figure 1. The yellow color of GO sheets remained after the incorporation of TRIS moieties, which also suggested that GO-TRIS retained the oxygen-containing groups so as to stabilize the corresponding aqueous solution.

**Figure 2:**
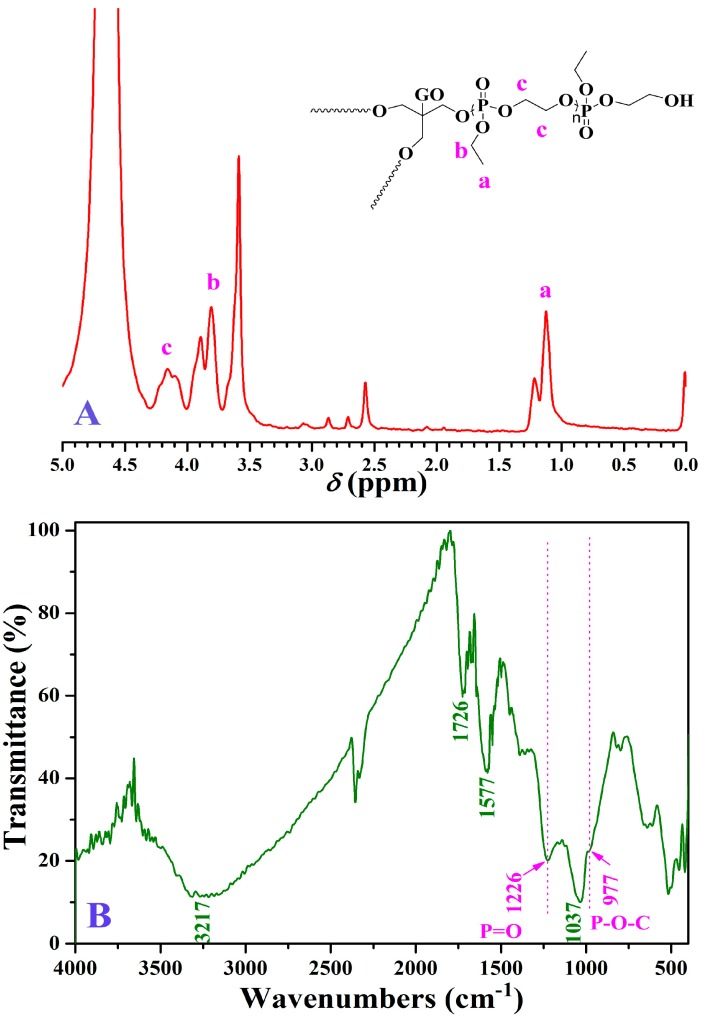
^1^H NMR (**A**) and Fourier transform infrared (FT-IR) (**B**) spectra of GO-TRIS-PEEP.

Thermo-responsive PEEP chains then grew from the fabricated hydroxyls on the surface through ROP. To ensure that there was no polymer chains adsorbed on the surface of GO, the obtained product was filtered through a 0.22 μm filter followed by washing with THF constantly, until the P content determined by elementary analysis was detected to be zero in the filtrate. ^l^H NMR and FT-IR analyses were employed to characterize the obtained product after ROP of EEP monomer for confirming GO functionalization and the presence of PEEP chains on the surface of GO. ^1^H NMR spectrum of the resulting GO-TRIS-PEEP in D_2_O is shown in Figure 2A and the peaks located at 1.12 (3H) and 3.81 (2H) ppm are attributed to the POCH_2_CH_3_ moiety in the side group of PEEP, respectively. On the other hand, the proton resonance signal of OCH_2_CH_2_ moiety in the backbone of PEEP is also seen at 4.16 ppm. Owing to the existence of the hydrophilic PEEP chains, GO-TRIS-PEEP might be well dispersed in D_2_O. GO-TRIS-PEEP showed relative strong bands at 3217, 1726, 1577 and 1037 cm^−1^ in FT-IR spectrum (Figure 2B), corresponding to stretching vibrations of O-H, C=O, C=C and C-O groups, respectively. The stretching vibration peaks attributed to P=O and P-O-C appear at 1226 and 977 cm^−1^ after ROP. These characteristic peaks are originated from polyphosphoester chains attached to the surface through ROP. These data clearly illustrate the successful ROP of EEP initiated by GO-TRIS.

**Figure 3:**
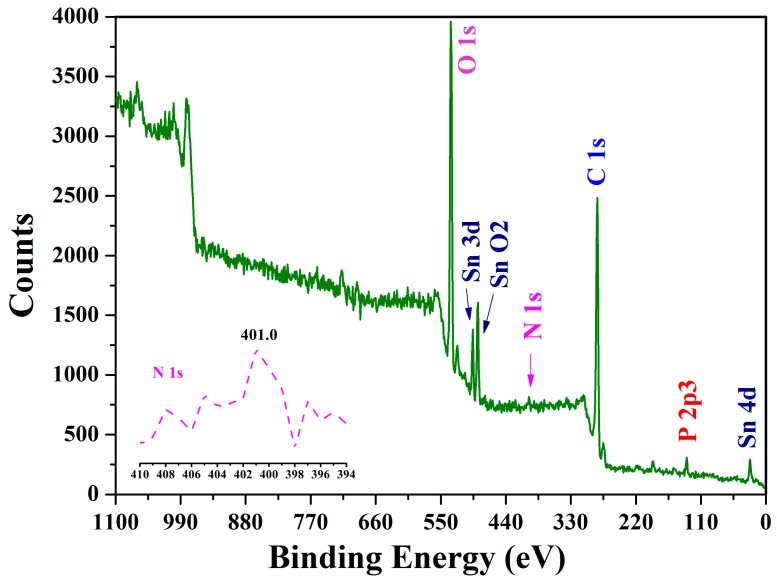
Survey XPS data for GO-TRIS-PEEP.

To further understand the effective functionalization of PEEP chains on GO sheets, XPS measurement was employed to provide qualitative analysis. GO-TRIS-PEEP shows a strong peak of C1s at a binding energy around 285 eV, a weak peak of N1s at around 401.0 eV and a peak at around 533 eV originating from O1s (Figure 3). Furthermore, a peak at around 134 eV is assigned to P2p3, which is resulted from PEEP chains. As the coordination between Sn of the catalyst and hydroxyls in the polymer chains [44], we also can observe the peaks of Sn4d around 24 eV, SnO2 around 487 eV and Sn3d around 497 eV. The molar contents of C, O, N and P are 65.66%, 30.44%, 0.98% and 1.89%, respectively. The weight percentage of grafted PEEP in GO-TRIS-PEEP is estimated to be 21.5 wt % from Equation (1).

PEEP% = [*c*_P_ × *A*_P_/∑(*c*_i_ × *A*_i_)] × (152/31) × 100% (1)

**Figure 4:**
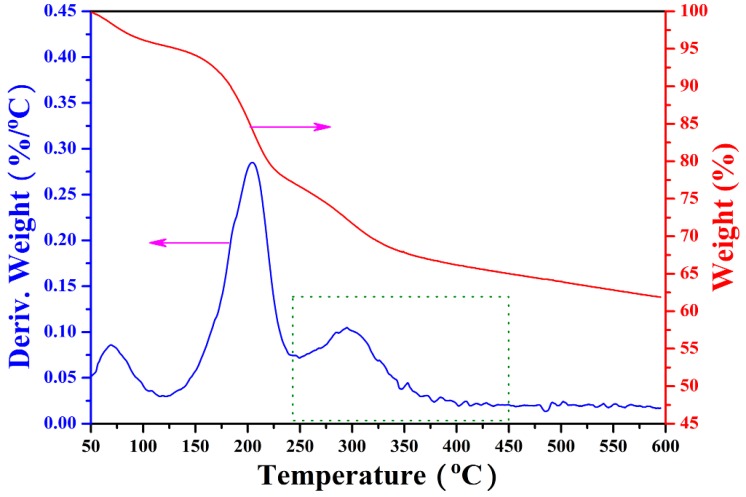
Thermogravimetric analysis (TGA)(red line) and differential thermal gravity (DTG) (blue line) curves of GO-TRIS-PEEP in N_2_.

TGA analysis of GO-TRIS-PEEP was performed to elucidate its thermal behavior and chemical composition. In comparison with the initial GO (Figure S4), the thermograph of the obtained GO-TRIS-PEEP (Figure 4) exhibits three major stages of decomposition in the temperature range of 50–600 °C. The first stage shows a low weight loss until 125 °C, corresponding to the loss of adsorbed water into its structure. The second stage from 125 °C to 250 °C can be assigned to the decomposition of oxygen-containing functionalities by evaporation of CO and CO_2_. The last weight loss occurs between 250 °C and 450 °C, indicating the degradation of PEEP segment. To further investigate the enhancement of thermal properties, differential thermal gravity (DTG) curve of GO-TRIS-PEEP is also plotted in Figure 4. The peaks in DTG curve correspond to the temperatures at maximum rate of weight loss (*T*_max_), and the outcome is entirely consistent with the TGA data. The quantity of PEEP chains attached to the surface of GO sheets is also determined from TGA. Figure 4 shows a 14.6 wt % (= 79.6 wt % − 65.0 wt %) weight loss at the temperature range from 250 °C to 450 °C, and thus, the amount of PEEP chains grafted onto GO sheets is about 14.6 wt %. Compared to GO, most of PEEP chains were exposed on the surface of GO-TRIS-PEEP, the measured phosphorus content by XPS might be higher than actual data. As a result, the XPS data (21.5%) is a little higher than that obtained from TGA (14.6%).

### 3.2.Surface Morphologies of GO-TRIS-PEEP Sheets

**Figure 5:**
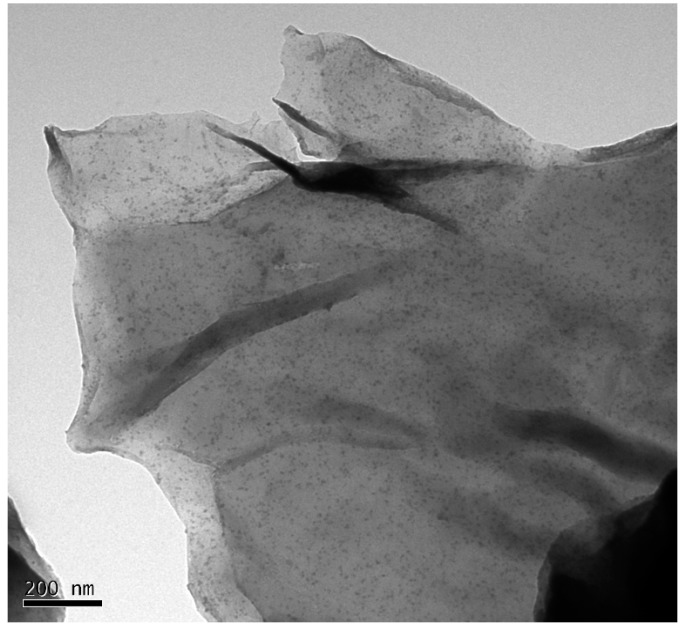
Transmission electron microscope (TEM) image of GO-TRIS-PEEP aqueous solutions with a concentration of 0.01 mg/mL using drop-casting method.

A TEM micrograph of the fabricated GO-TRIS-PEEP is presented in Figure 5. It can be observed that the lamellar morphology of GO is preserved after ROP of EEP monomer, and the individual sheets have sizes of about 1 μm. Compared to GO (Figure S5), new dark dots appear because of the tangled mess formed by the grafted PEEP chains. Previous studies on GO have identified that carboxyls are at the periphery of sheets, while the oxygen-containing functionalities such as hydroxyl and epoxy group are on the basal plane [45,46]. As hydroxyls were used as initiating sites to trigger the polymerization of EEP monomer via surface-initiated ROP, the blackish polymeric chains spread evenly on the surface of the GO sheets, which is consistent with previous reports [47,48]. The grafted polymeric chains also destroyed the ordered π-π stacking of graphitic basal planes so that functionalized GO sheets appeared to have better dispersion and exfoliation in methanol solution (Figure S6).

**Figure 6:**
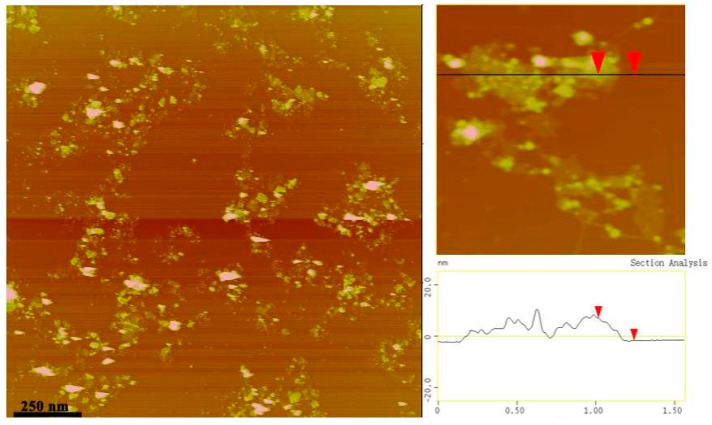
Atomic force microscopy (AFM) images of GO-TRIS-PEEP.

AFM provides a direct method to characterize the surface morphology and thickness of GO-TRIS-PEEP sheets. As shown in Figure 6, GO-TRIS-PEEP sheets have an average in-plane size with several hundreds of nanometers. The surfaces of GO-TRIS-PEEP sheets are very rough compared to those of pristine GO sheets (Figure S7) and the thickness of GO-TRIS-PEEP sheets is measured to be approximately 10 nm from the average height profiles, which is much higher than that of pristine GO sheets. Both the rough surfaces and higher thickness of GO-TRIS-PEEP sheets further demonstrate the successful polymeric grafting [49,50,51].

### 3.3. Thermo-Responsive Behavior of GO-TRIS-PEEP

**Figure 7:**
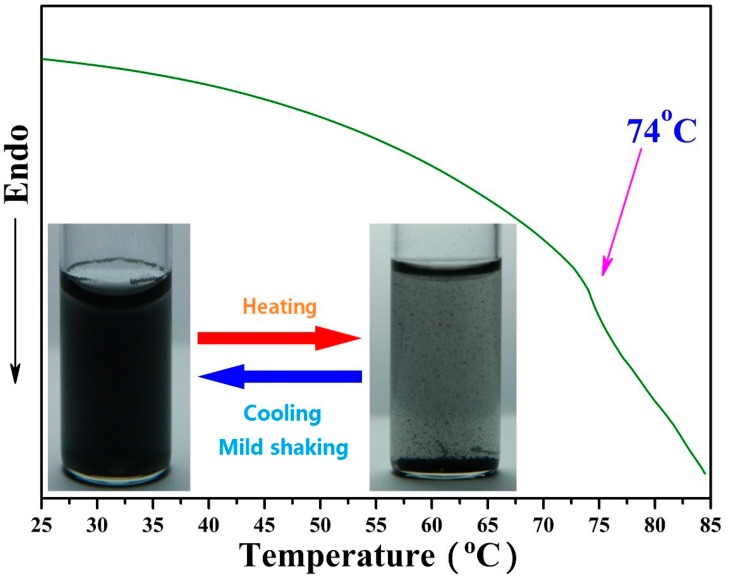
DSC curve of GO-TRIS-PEEP with a heating rate of 10 °C/min in N_2_, the inset shows the thermo-stimuli reversible phase behavior of GO-TRIS-PEEP.

As it is well known, PEEP has a phase transition behavior in water and form aggregates by making the solution turbid when the temperature is above its low critical solution temperature (LCST) [32,38]. The LCST behavior of PEEP is attributed to a balance between hydrophilic and hydrophobic interactions and the resulting hydrogen-bonding interactions between water molecules and the polymeric chains. It is one of the basic physical properties of thermo-responsive water-soluble polymers [52]. As expected, this thermal behavior in aqueous solution was well retained after PEEP was covalently bound to GO sheets (see the inset in Figure 7). In a typical experiment, we found that GO-TRIS-PEEP with a concentration of 0.5 mg/mL at 25 °C formed a stable homogeneous solution. If not heated, the homogeneous solution could last more than one month. When the solution was heated to 40 °C, GO-TRIS-PEEP began to aggregate. Further raising the temperature, GO-TRIS-PEEP would precipitate completely from the solution in a few minutes. When the mixture was cooled down to 25 °C, and then mildly shaken for several minutes, a homogeneous solution recovered again. This precipitation phenomenon was not observed for GO aqueous dispersion even if the temperature rose to 90 °C (Figure S8), which demonstrated that PEEP segments on the surface of GO-TRIS-PEEP take account for the above- mentioned thermal responsibility. The hydrophilic/hydrophobic phase transition of PEEP-grafted GO sheets should show exothermic or endothermic effects. The change of enthalpy during phase transition can be measured by DSC. The DSC curve (Figure 7) exhibits a small endothermic peak at ~74 °C, which indicates a phase transition process. Considering that GO-TRIS-PEEP nanocomposite begins to aggregate at 40 °C, the phase transition process should be gradual in a wide temperature range. It might be because the abundant oxygen-containing functionalities on the surface increased the hydrophilicity of GO-TRIS-PEEP. Therefore, it is not appropriate to provide a certain temperature as the LCST.

## 4. Conclusions

In summary, we have successfully modified GO with the thermo-responsive PEEP chains via the grafting-from approach. To the best knowledge, this is the first example of PPE-Based GO/polymer nanocomposite via covalent modification. The structure of the resulting GO-TRIS-PEEP nanocomposite was well studied by various characterizations to prove the change on functionalization. Moreover, GO-TRIS-PEEP nanocomposite shows good temperature responsive behavior as a result of a conformation change in PEEP chains grafted on the surface of GO. Our work provides a potent strategy for the design and synthesis of novel GO/stimuli- responsive polymer nanocomposites, which exhibit great application potential in biological and medical as well as interdisciplinary fields.

## Supplementary Materials

The following are available online at http://www.mdpi.com/2079-4991/9/2/207/s1, Figure S1: FT-IR spectra of natural graphite powder and graphene oxide, Figure S2: X-ray diffraction patterns of natural graphite powder and graphene oxide, Table S1: Elementary analysis of natural graphite and graphene oxide, Figure S3: Survey XPS data for graphene oxide, the inset curve indicates that there is no N1s peak in XPS of graphene oxide, Figure S4: TGA (black) and DTG (blue) curves of graphene oxide, Figure S5: TEM images of graphene oxide with different scale bars in length, (A) 1 μm and (B) 200 nm, Figure S6: AFM images of graphene oxide, Figure S7: AFM images of graphene oxide, Figure S8: Photographs of graphene oxide aqueous solutions in different temperature with a concentration of ~0.5 mg/mL.
