# Supplementary material for: Thermo-Responsive Graphene Oxide/Poly(Ethyl Ethylene Phosphate) Nanocomposite via Ring Opening Polymerization
Source: Nanomaterials (Basel). 2019 Feb 5;9(2):207. doi: 10.3390/nano9020207 (PMC6409759; doi:10.3390/nano9020207)
Supplement: Supplementary file 1 [file nanomaterials-09-00207-s001.pdf]

nanomaterials

Supporting Information for

Thermo-responsive Graphene Oxide/Poly(ethyl ethylene phosphate) Nanocomposite via Ring Opening Polymerization

Xue Jiang,[1,2] *Guolin Lu*,[2] *Xiaoyu Huang*,[2,*] *Yu Li*,[1] *Fangqi Cao*,[1]

Hong Chen,[1] and *Wenbin Liu*,[1,*]

[1] Shanghai Key Laboratory of Crime Scene Evidence, Shanghai Research Institute of Criminal Science and Technology, Zhongshan North No 1 Road, Shanghai 200083, People's Republic of China

[2] Key Laboratory of Synthetic and Self-Assembly Chemistry for Organic Functional Molecules, Shanghai Institute of Organic Chemistry, Chinese Academy of Sciences, 345 Lingling Road, Shanghai 200032, People's Republic of China

* To whom correspondence should be addressed, E-mail: wbliu1981@163.com (Tel: +86-21-22028361), xyhuang@sioc.ac.cn (Tel: +86-21-54925310, Fax: +86-21-64166128).

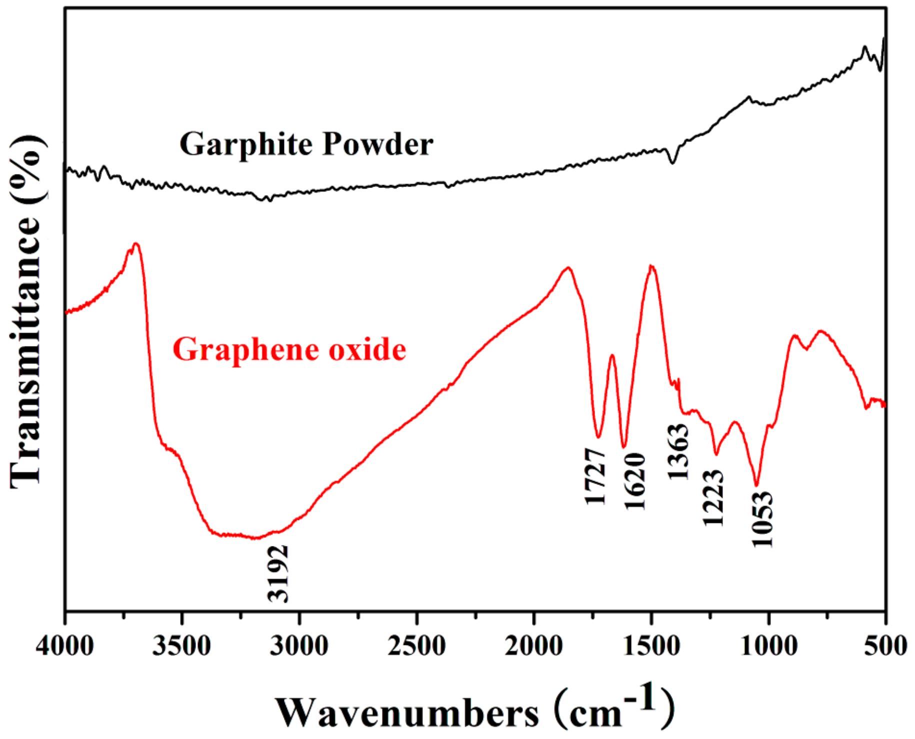

Figure S1. FT-IR spectra of natural graphite powder and graphene oxide.

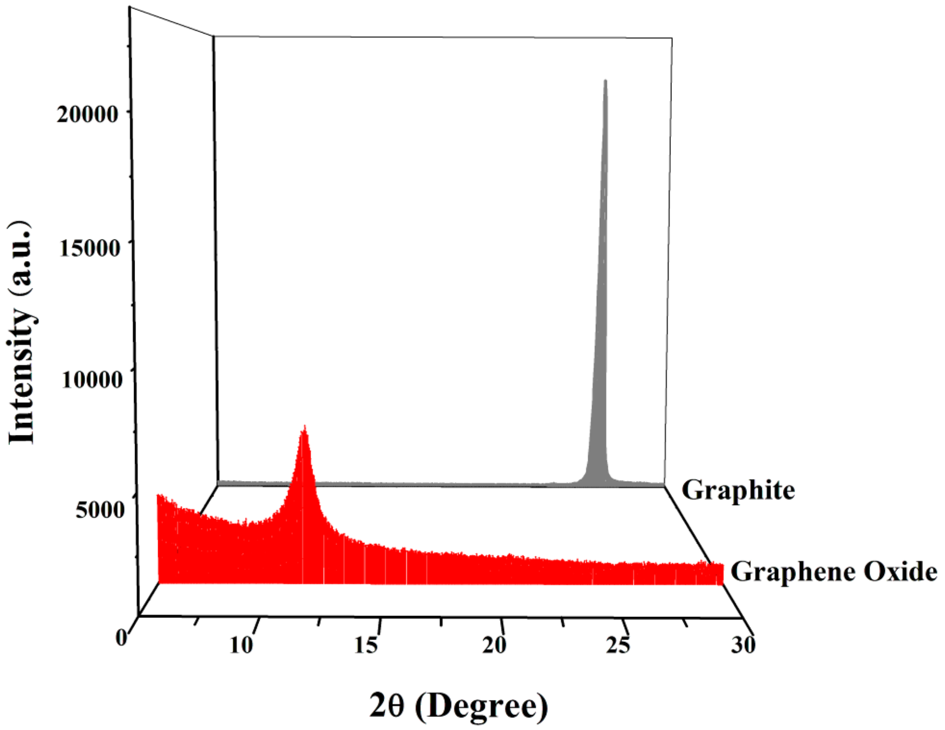

Figure S2. X-ray diffraction patterns of natural graphite powder and graphene oxide.

Table S1. Elementary analysis of graphite and graphene oxide

Sample	Elemental Analysis (wt%)	
	C	O
Nature graphite	100	<0.5
Graphene oxide	43.53	52.89

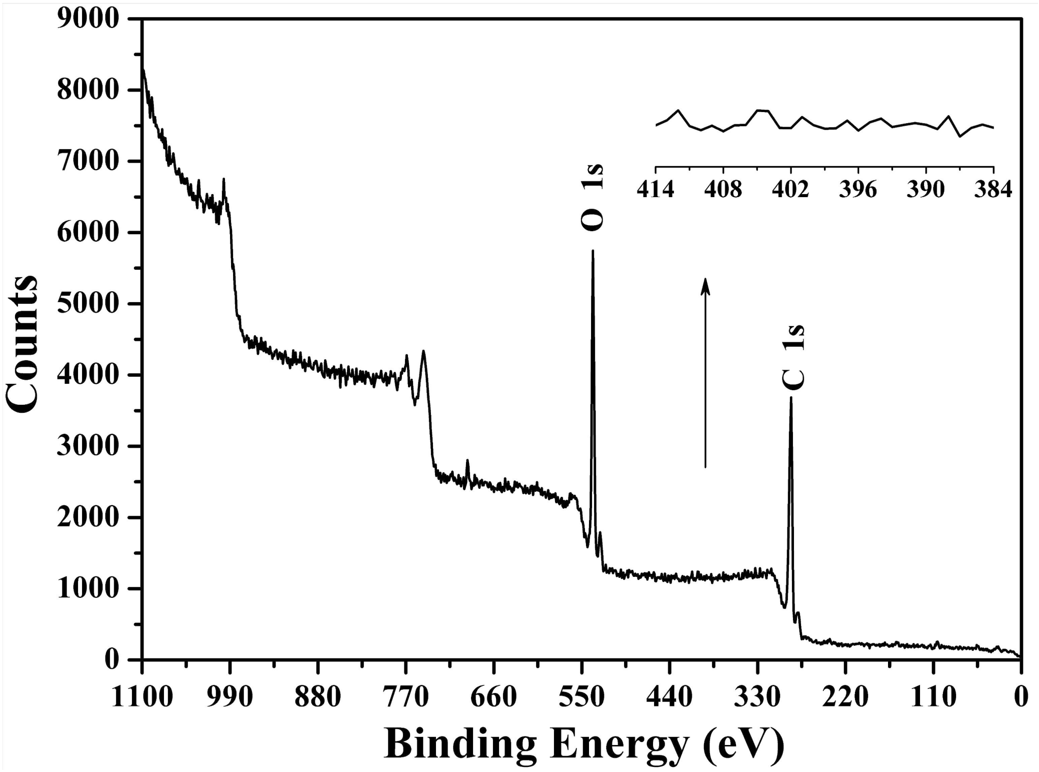

Figure S3. Survey XPS data for graphene oxide, the inset curve indicates that there is no N1s spectrum in XPS of graphene oxide.

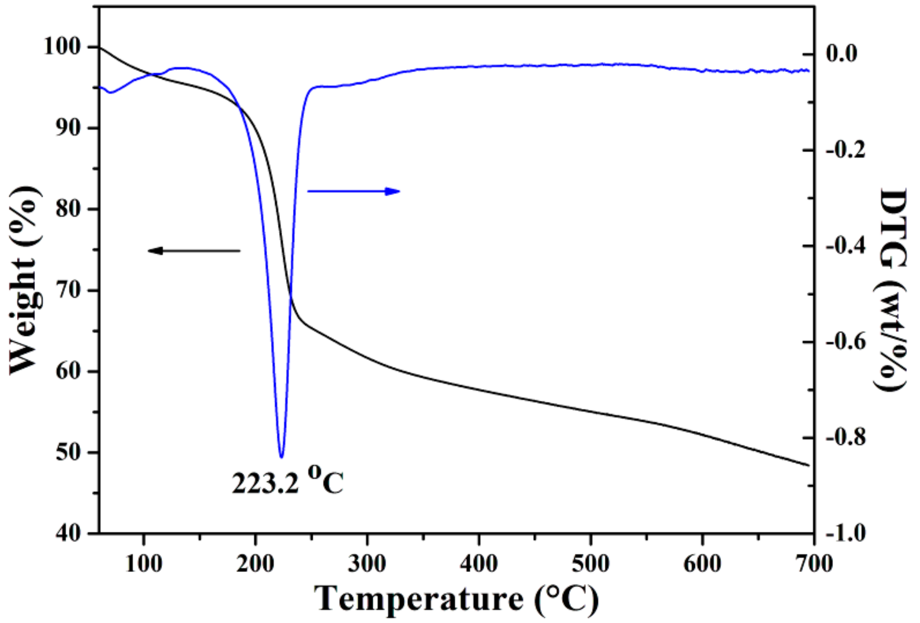

Figure S4. TGA (black) and DTG (blue) curves of graphene oxide.

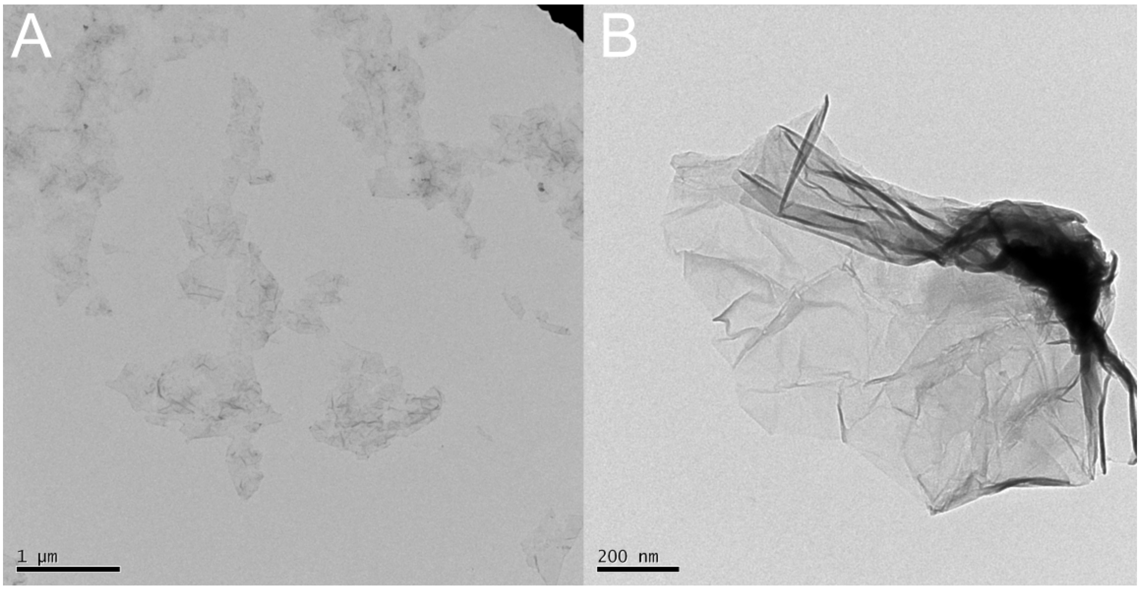

Figure S5. TEM images of graphene oxide with different scale bars in length, (A) 1µm and (B) 200 nm.

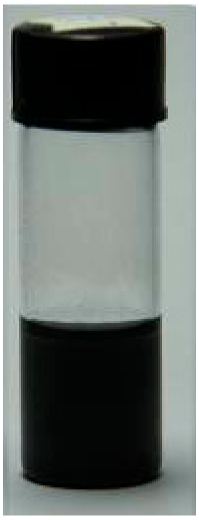

Figure S6. Dispersion of GO-TRIS-PEEP in methanol with a concentration of about 0.5 mg/mL.

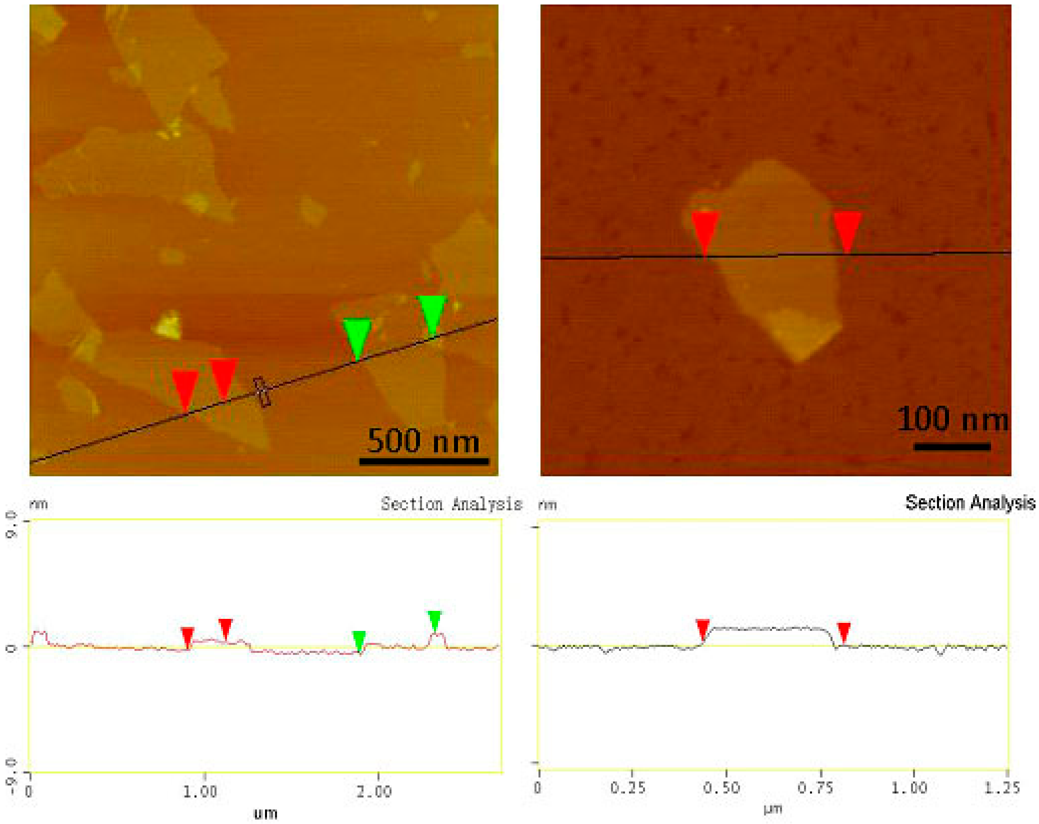

Figure S7. AFM images of graphene oxide.

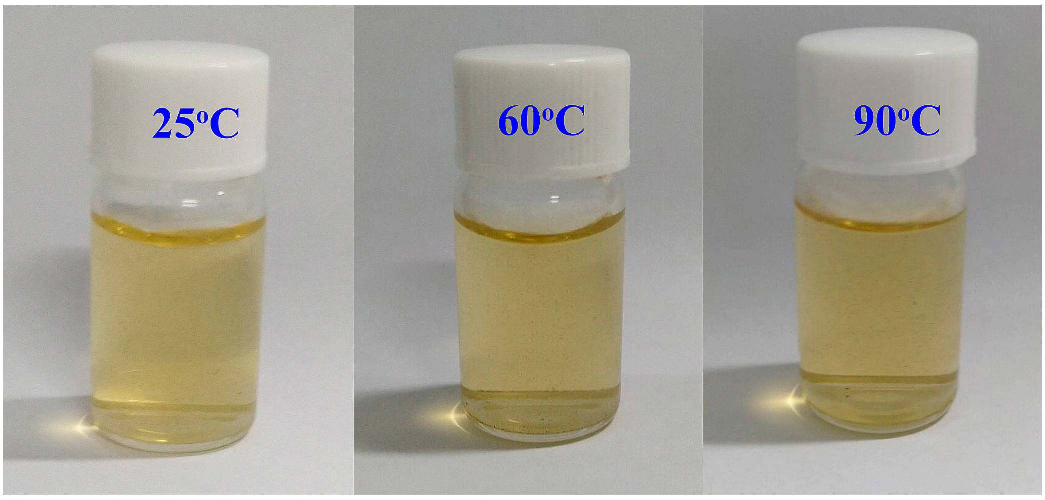

Figure S8. The photographs of graphene oxide aqueous solutions in different temperature with a concentration of 0.5 mg/mL
